# Onset of pulmonary Epstein–Barr virus‐positive diffuse large B‐cell lymphoma in a patient with silicosis

**DOI:** 10.1111/1759-7714.14250

**Published:** 2021-11-25

**Authors:** Ryosuke Ogata, Hiroshi Soda, Yasuhiro Tanaka, Hiroaki Senju, Midori Shimada, Koki Yamashita, Shota Nakashima, Asuka Umemura, Masataka Yoshida, Takuya Hara, Saeko Jinnai, Keisuke Iwasaki, Yuichi Fukuda, Hiroyuki Yamaguchi, Hiroshi Mukae

**Affiliations:** ^1^ Department of Respiratory Medicine Sasebo City General Hospital Nagasaki Japan; ^2^ Precision Medicine Center Sasebo City General Hospital Nagasaki Japan; ^3^ Department of Pathology Sasebo City General Hospital Nagasaki Japan; ^4^ Department of Infection Control and Prevention Sasebo City General Hospital Nagasaki Japan; ^5^ Department of Respiratory Medicine Nagasaki University Graduate School of Biomedical Sciences Nagasaki Japan

**Keywords:** Epstein–Barr virus, immune cells, lymphoma, silicosis

## Abstract

How Epstein–Barr virus (EBV)‐positive diffuse large B‐cell lymphoma (DLBCL) occasionally occurs following chronic inflammation remains to be elucidated. The case of a 57‐year‐old man who developed pulmonary EBV‐positive DLBCL from underlying silicosis lesions is presented. Immunohistochemical examination of the resected silicosis lesions showed predominant helper T cells and M1/M2 macrophages, with a lack of B cells, regulatory T cells, and resident memory T cells. Two years later, EBV‐positive DLBCL emerged unexpectedly from the silicosis. The imbalance of the immune cells in the microenvironment, at least in part, may help explain how chronic inflammation contributes to EBV‐positive DLBCL.

## Graphical abstract

The underlying silicosis lesions showed predominant Th cells and M1/M2 macrophages, with a lack of B cells, Treg cells, and T_RM_ cells. The imbalance of the immune cells in the microenvironment, at least in part, may help to explain how chronic inflammation contributes to EBV‐positive diffuse large B‐cell lymphoma.

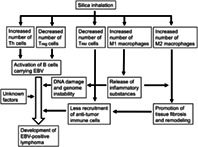

## INTRODUCTION

Diffuse large B‐cell lymphoma (DLBCL) associated with chronic inflammation is categorized as a subtype of Epstein–Barr virus (EBV)‐positive DLBCL.^1^ This disease occasionally develops from long‐term inflammation due to pyothorax or artificial implants.^2^ In the presence of inflammation, immune cells release inflammatory substances that could lead to DNA damage and genome instability, causing a predisposition to malignant tumors. However, little is known about the immunological mechanisms of developing DLBCL during the process of chronic inflammation.^3^ To the best of our knowledge, this is the first report of a case of pulmonary EBV‐positive DLBCL arising from silicosis examining the underlying immune microenvironment of the silicosis immunohistochemically.

## CASE REPORT

**FIGURE 1:**
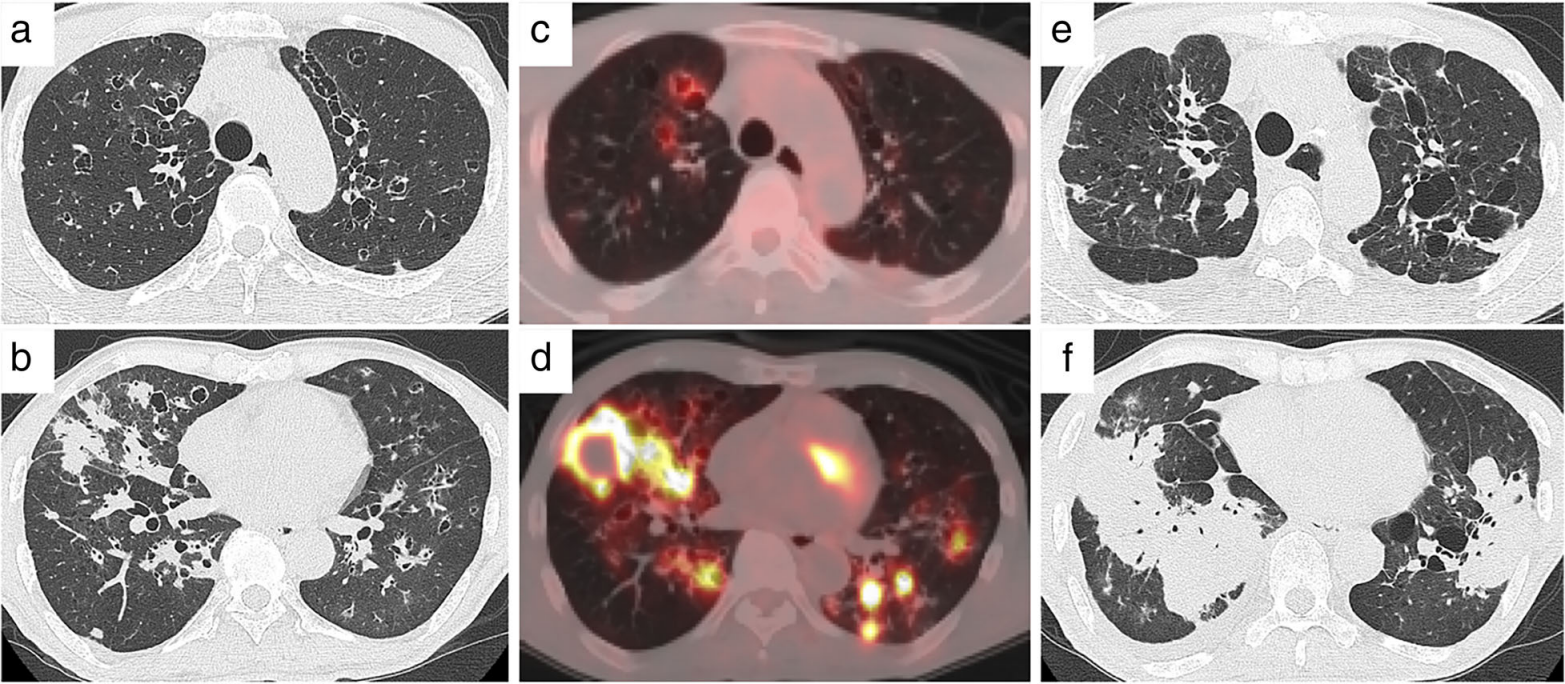
Chest radiological findings in a patient with silicosis. Chest computed tomography scans at the first visit show (a) cystic lesions and bronchiectasis at the upper lobes, and (b) multiple nodules at the right middle and bilateral lower lobes. Positron emission tomography shows high uptake of ^18^F‐fluorodeoxyglucose (c) into the cystic lesions and (d) the multiple nodules. Two years after the first visit, chest computed tomography scans show (e) the deterioration of cystic lesions and bronchiectasis at the upper lobes and (f) the emergence of several masses at the lower lobes

An asymptomatic 57‐year‐old male ex‐smoker was referred to our department for evaluation of multiple lung nodules on chest radiography. He had produced Japanese‐style floor mats made of straw and rush, called *tatami* in Japanese. He had potentially inhaled silica dust during its manufacturing process. Physical examination was unremarkable for lung auscultation and lymphadenopathy. Laboratory tests showed slightly elevated serum levels of C‐reactive protein at 2.1 ng/ml (reference value <0.3 ng/ml). The circulating antibodies for EBV and human immunodeficiency virus were not examined. Chest computed tomography showed multiple solid nodules, bronchiectasis, and cystic lesions in both lungs, with no enlargement of mediastinal lymph nodes (Figure 1a,b). Positron emission tomography showed high uptake of ^18^F‐fluorodeoxyglucose only into the nodular and cystic lesions (Figure 1c,d). Although the nodules were suspected to be metastatic lung cancer, transbronchial lung biopsy failed to establish the diagnosis. The patient then underwent thoracoscopic lung biopsy of the nodules at the right lower lobe.

**FIGURE 2:**
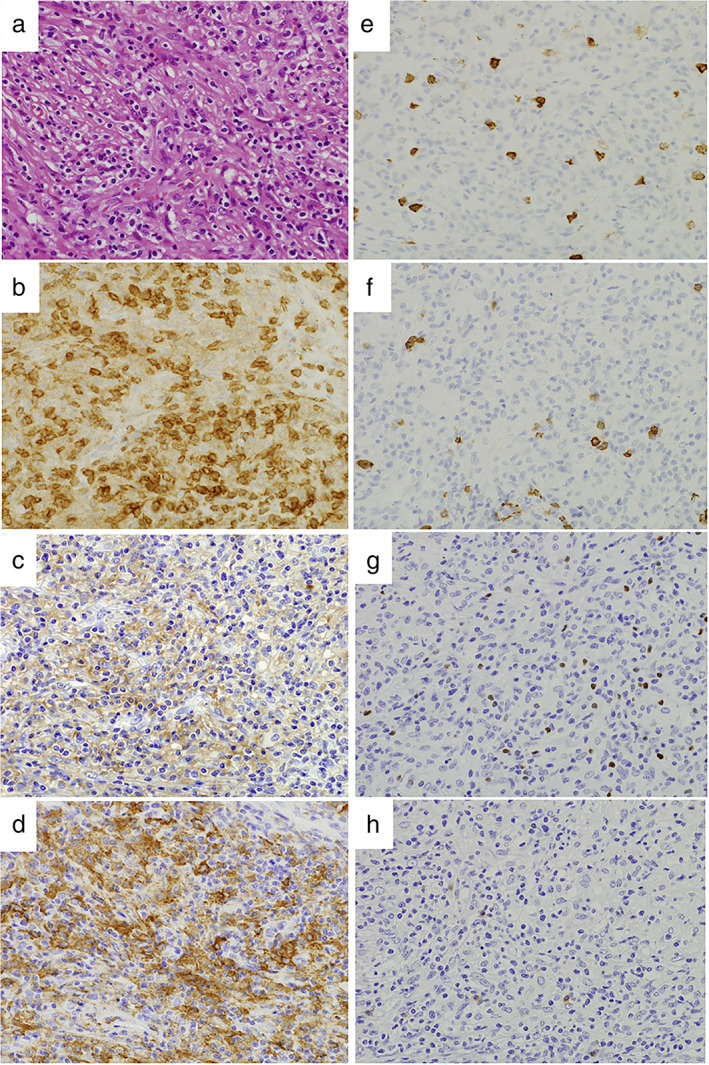
Photomicrographs of thoracoscopic lung biopsy specimen taken from a patient with silicosis (original magnification 40×). (a) Fibrosis associated with lymphohistiocytic infiltration is observed (hematoxylin & eosin stain). The immunohistological study indicates (b) the predominance of CD4^+^ helper T cells, (c) CD11c^+^ M1 macrophages, and (d) CD163^+^ M2 macrophages. In contrast, it shows (e) the lack of CD8^+^ cytotoxic T cells, (f) CD20^+^ B cells, (g) FOXP3^+^ regulatory T cells, and (h) CD103^+^ resident memory T cells

Pathologically, the resected specimens showed that silica was deposited within the nodules, which were eventually diagnosed as silicosis. The fibrotic nodules also included many CD3^+^ lymphocytes and CD68^+^ macrophages. The lymphocytes consisted mainly of CD4^+^ helper T cells (Figure 2). There was little infiltration of CD20^+^ B cells, FOXP3^+^ regulatory T cells, CD8^+^ cytotoxic T cells, CD103^+^ resident memory T cells, and CD56^+^ natural killer cells. The macrophages were composed equally of CD11c^+^ M1 phenotypes and their CD163^+^ M2 counterpart. EBV‐encoded small RNA (EBER) was not seen in these immune cells.

The patient regularly visited a medical clinic and our hospital. The silicosis had an uneventful course without the specific treatment for 2 years. Thereafter, pulmonary nodules newly emerged and increased in size at both lower lobes (Figure 1e,f). The patient experienced fever and dyspnea. During the treatment with antibiotics, his condition was gradually complicated by right refractory pneumothorax, pulmonary infection by *Aspergillus fumigatus*, and cytomegalovirus colitis. Despite intensive treatment, he died of multiple organ failure 3 years after the first visit. The patient's guardian permitted the autopsy of the patient.

**FIGURE 3:**
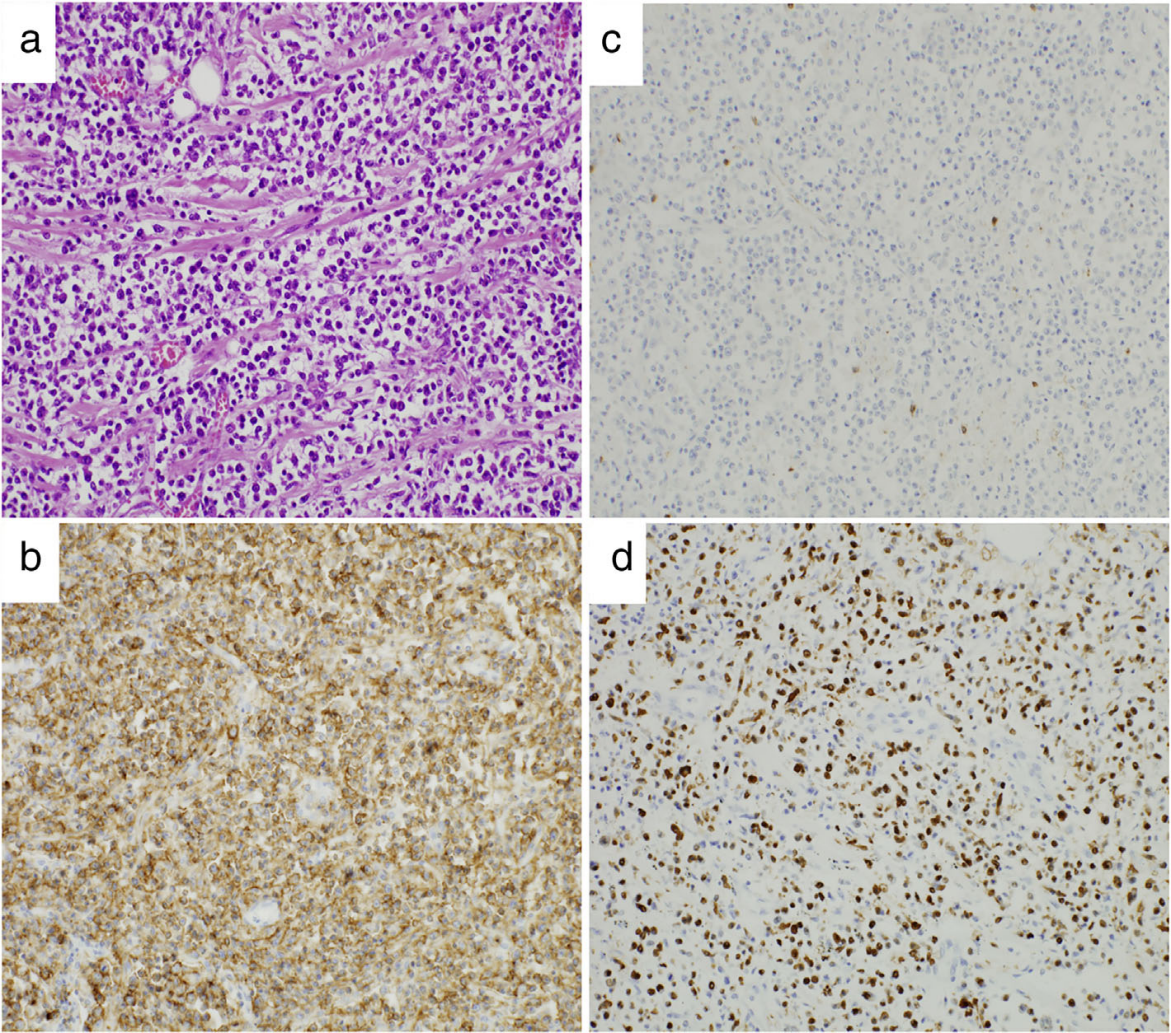
Photomicrographs of the autopsy specimen taken from a patient with Epstein–Barr virus‐positive diffuse large B‐cell lymphoma associated with silicosis (original magnification 40×). (a) Lymphoma cells are seen (hematoxylin & eosin stain). The immunohistological study shows (b) lymphoma cells positive for CD20, (c) negative for CD3, and (d) positive for Epstein–Barr virus small RNA

On immunohistochemical examination of the autopsy specimens, the bilateral lung tumors were composed of CD20^+^ atypical lymphocytes positive for EBER (Figure 3). The atypical lymphocytes were weakly positive for MUM1 and negative for CD10 and Bcl‐6. No atypical lymphocytes were observed in the other organs. Taken together, the tumors were diagnosed as pulmonary EBV‐positive DLBCL showing the activated B cell phenotype. The antibody clones used were as follows: Bcl‐6 (LN22), CD3 (F7.2.38), CD4 (4B12), CD8 (4B11), CD10 (56c6), CD11c (5D11), CD20 (L‐26), CD56 (123C3), CD68 (KP‐1), CD103 (Ab129202), CD163 (10D6), EBER (Leica‐microsystems, catalog #PB0589), FOXP3 (236A/E7), and MUM1 (EAU32).

## DISCUSSION

This case highlights two important findings. First, the underlying silicosis lesions included predominant helper T cells with a lack of B cells, regulatory T cells, and resident memory T cells. Consistent with previous studies,^4^, ^5^ the inhalation of silica increased the activity of helper T cells, whereas it decreased the immunosuppressive function of regulatory T cells. The imbalance between helper T cells and regulatory T cells may initiate the transformation of EBV‐infected B cells. A retrospective study reported that the risk of EBV‐positive Hodgkin lymphoma depended on the elevated counts of CD4^+^ T cells after antiretroviral therapy in patients with human immunodeficiency virus infection.^6^ Moreover, the lack of resident memory T cells may have decreased the recruitment of cytotoxic T cells against EBV‐transformed B cells.^7^

The second important finding in this case is that M1/M2 macrophages were increased in the silicosis lesions.^4^ Macrophages can differentiate into either the M1 or M2 functional subtypes; M1 macrophages release inflammatory substances, whereas M2 macrophages suppress the inflammatory reaction. In the present case, positron emission tomography showed high uptake of ^18^F‐fluorodeoxyglucose into the cystic and nodular lesions, suggesting the presence of inflammation in the lungs. The inflammatory substances could lead to DNA damage and genome instability in the adjacent cells, resulting in the predisposition to malignant transformation.^8^, ^9^ The continuous exposure to silica encouraged M1 macrophages to initiate the fibrotic process, where M2 macrophages promoted the proliferation of fibroblasts and remodeling of the lung structures.^10^ Fibrosis accompanied by bronchiectasis and multiple cystic lesions may have prevented immune cells from entering the malignant lesions.

A limitation of this report is that the findings are obtained only in one case. However, EBV‐positive DLBCL has been frequently reported in patients with chronic inflammation, such as pyothorax, metallic implants, chronic skin ulcers, chronic osteomyelitis, and breast implants.^3^ The lymphoma cells had a complex karyotype and hypermutated genes.^11^ In contrast, the lymphoma cells produced immunosuppressive cytokines and downregulated the expression of HLA class I.^3^ Unknown factors may be involved in the onset of EBV‐positive DLBCL. Further investigations are required to clarify how EBV‐positive DLBCL occurs during the process of chronic inflammation.

In conclusion, predominant helper T cells and M1/M2 macrophages with a lack of regulatory T and resident memory T cells could contribute, at least in part, to the development of EBV‐positive DLBCL from silicosis. These findings may provide a clue for understanding how EBV‐positive DLBCL emerges following chronic inflammation.

## CONFLICT OF INTEREST STATEMENT

No authors report any conflicts of interest. Written, informed consent for the publication of this case report was obtained from the patient's guardian.
